# Supplementary material for: Deconvolution and phylogeny inference of diverse variant types integrating bulk DNA-seq with single-cell RNA-seq
Source: Bioinform Adv. 2025 Sep 29;5(1):vbaf234. doi: 10.1093/bioadv/vbaf234 (PMC12571511; doi:10.1093/bioadv/vbaf234)
Supplement: vbaf234_Supplementary_Data [file vbaf234_supplementary_data.zip › Bristy Bioinf Adv Supplement.pdf]

Supplementary Material for Bristy and Schwartz – Deconvolution and Phylogeny Inference of Diverse Variant Types Integrating Bulk DNA-seq with Single-cell RNA-seq

Supplementary Methods

Explanatory figures

Figure S1 shows a toy example of the inputs and outputs of TUSV-INT. Figure S2 shows the schematic diagram of TUSV-INT.

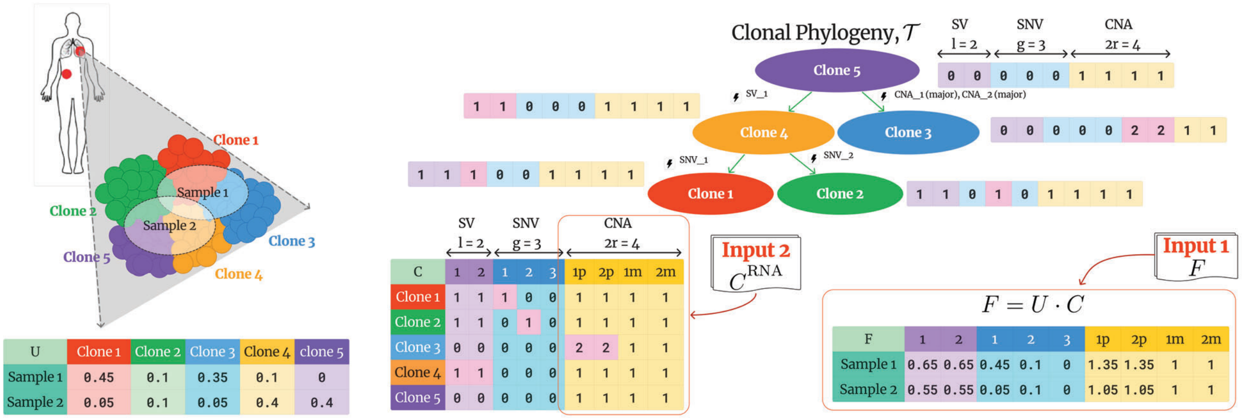

U	Clone 1	Clone 2	Clone 3	Clone 4	clone 5
Sample 1	0.45	0.1	0.35	0.1	0
Sample 2	0.05	0.1	0.05	0.4	0.4

C	1	2	1	2	3	1p	2p	1m	2m
Clone 1	1	1	1	0	0	1	1	1	1
Clone 2	1	1	0	1	0	1	1	1	1
Clone 3	0	0	0	0	0	2	2	1	1
Clone 4	1	1	0	0	0	1	1	1	1
Clone 5	0	0	0	0	0	1	1	1	1

F	1	2	1	2	3	1p	2p	1m	2m
Sample 1	0.65	0.65	0.45	0.1	0	1.35	1.35	1	1
Sample 2	0.55	0.55	0.05	0.1	0	1.05	1.05	1	1

Fig. S1. A toy-example of TUSV-INT's inputs. The two inputs are the bulk mixture copy-number matrix F and single cell copy number alterations C^{RNA} coming from the single-cell RNA-seq. Given F and C^{RNA}, the outputs of TUSV-INT are clonal mixture fraction matrix U, variant copy number matrix C and the clonal phylogeny $\mathcal{T}$.

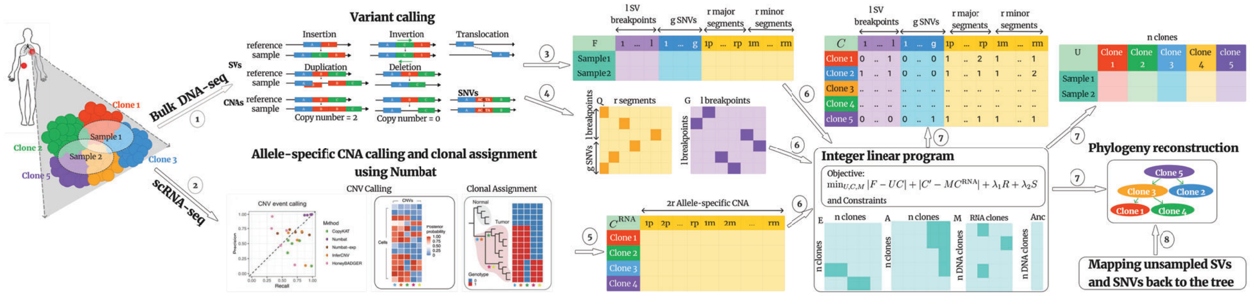

Fig. S2. A graphical overview of TUSV-int. Multisample bulk DNA-seq and scRNA-seq data are collected from the same patient. (1) Mixture copy numbers for SNV positions, SV breakpoints, and allele-specific CNA segments are derived from the bulk DNA-seq samples. (2) Allele-specific CNAs are independently inferred from the scRNA-seq data. (3) From the bulk data, we compute the mixture fraction matrix F, the SNV/SV-to-CNV segment mapping matrix Q, and the SV breakpoint pairing matrix G. (4) The scRNA-seq data are used to construct the clonal copy number matrix C^{RNA}, which captures copy number states across inferred clones. (5) The matrices F, Q, G, and C^{RNA} are then used as input to our integer linear programming (ILP) framework, which jointly infers the tumor phylogeny and subsamples variants to optimize the objective function. (6) The ILP yields the clonal phylogeny along with the clonal frequency matrix U and the clonal copy number matrix C. (7) In the final step, unsampled SNVs and SVs are mapped back onto the inferred phylogeny to complete the clonal reconstruction.

Integer linear program formulation

In this section, we elaborate on the constraints of the ILP described in the main paper. We have described the constraints while optimizing for the $U, C, M,$ and C' matrices in the main text. In the main text, we described the coordinate descent algorithm to first fix $M, C,$ and C' matrices and estimate U and then fix the U matrix to estimate $M, C,$ and C'. Since the relation between these matrices is linear, we can estimate these in the same coordinate descent step. While estimating these matrices, we require additional constraints described below.

Estimating clonal mixture fraction matrix U

This section is omitted because it is fully described in the main text.

Estimating M and C'

This section is also omitted because it is fully described in the main text.

Phylogenetic and ancestry constraints

We represent the phylogenetic tree, $\mathcal{T}$ with the help of an $n \times n$ edge matrix E and an $n \times n$ ancestor matrix A. E and A help in describing the parent-child relationships of the nodes as well as establishing copy number constraints on the C matrix. The constraints on the E and A matrix are described in the main text. We impose two additional constraints on A to ensure there is no cycle in the tree.

$$A_{i,j} + A_{j,i} \leq 1 \quad \forall i,j \in \{1,\dots,n\} \tag{S1}$$

$$A_{i,i} \leq 0 \quad \forall i \in \{1,\dots,n\} \tag{S2}$$

Copy number constraints and phylogenetic cost

As stated in the main text, we assume that the root clone is diploid, i.e., has diploid copy number segments and no SNVs or SVs (Eqn. 18, 19, 20). To compute the phylogenetic cost R, we introduce auxiliary variables and additional constraints as follows:

For each edge $E_{i,j}$, we define two auxiliary variables $x^1_{i,j,k}$ and $x^2_{i,j,k}$ to represent the absolute copy number differences for the two alleles of segment k:

$$0 \leq x^1_{i,j,k} \leq c_{\max} \cdot E_{i,j} \qquad \forall i,j \in \{1,\cdots,n\}, k \in \{1,\cdots,r\} \tag{S3}$$

$$0 \leq x^2_{i,j,k} \leq c_{\max} \cdot E_{i,j} \qquad \forall i,j \in \{1,\cdots,n\}, k \in \{1,\cdots,r\} \tag{S4}$$

To ensure $x^1_{i,j,k} = |C_{i,\ell+g+k} - C_{j,\ell+g+k}|$ when $E_{i,j} = 1$, we impose the following constraints:

$$x^1_{i,j,k} \geq C_{i,\ell+g+k} - C_{j,\ell+g+k} - c_{\max}(1 - E_{i,j}) \tag{S5}$$

$$x^1_{i,j,k} \geq -C_{i,\ell+g+k} + C_{j,\ell+g+k} - c_{\max}(1 - E_{i,j}) \tag{S6}$$

Similarly, to define $x^2_{i,j,k} = |C_{i,\ell+g+r+k} - C_{j,\ell+g+r+k}|$ when $E_{i,j} = 1$, we impose:

$$x^2_{i,j,k} \geq C_{i,\ell+g+r+k} - C_{j,\ell+g+r+k} - c_{\max}(1 - E_{i,j}) \tag{S7}$$

$$x^2_{i,j,k} \geq -C_{i,\ell+g+r+k} + C_{j,\ell+g+r+k} - c_{\max}(1 - E_{i,j}) \tag{S8}$$

These constraints ensure that the auxiliary variables have the absolute copy number differences only when an edge exists, and are set as zero otherwise.

Finally, we compute the phylogenetic cost R as the sum of copy number differences across all segments and edges:

$$\rho_{i,j} = \sum_{k=1}^{r} \left(x^1_{i,j,k} + x^2_{i,j,k} \right) \tag{S9}$$

$$R = \sum_{i=1}^{n} \sum_{j=1}^{n} \rho_{i,j} \tag{S10}$$

Dollo Phylogeny on breakpoints and SNVs

We define a binary indicator variable $\hat{x}$ to identify integers that are greater than zero for each integer variable x. For this, we follow Eaton et al. (Eaton et al., 2018) and Fu et al.'s (Fu et al., 2022) formulation, where,

$$\hat{x} = \begin{cases} 1 & \text{if } x > 0 \\ 0 & \text{otherwise} \end{cases} \tag{S11}$$

Let x_b be the bth bit of x and the maximum value x can take is $x_{\max}$. Then,

$$x = \sum_{b=0}^{\lfloor \log_2 x_{\max} \rfloor + 1} 2^b \cdot x_b$$

Now, to linearly define $\hat{x}$, we say that if any bit of x is one, then we set $\hat{x}$ as 1. For this we impose the following constraint.

$$0 \leq x_b \leq \hat{x} \leq \sum_{i=0}^{\lfloor \log_2 x_{\max} \rfloor + 1} x_i \tag{S12}$$

We use these binary variables to define the Dollo phylogeny constraints. Specifically, we impose constraints to ensure that if a breakpoint or SNV appears along an edge, we are able to detect it. Let $y_{i,j,b}$ be an auxiliary variable, which helps us define the matrix W, where $W_{i,j,b}$ denotes whether breakpoint or SNV b appears along the edge i, j. We use the following two equations to define it:

$$y_{i,j,b} = 2 + \hat{C}_{i,b} - \hat{C}_{j,b} - E_{i,j} \tag{S13}$$

$$W_{i,j,b} = 1 - \hat{y}_{i,j,b} \tag{S14}$$

Finally, to impose that a breakpoint or SNV is gained only once along the branches of the tree, we impose:

$$\sum_{i=1}^{n}\sum_{j=1}^{n} W_{i,j,b} = 1 \quad \forall b \in \{1, \cdots, l+g\} \tag{S15}$$

The loss of a breakpoint or SNV, however, is treated as a degenerate case of the loss of a segment, which is modeled by copy number loss. Similarly, we also constrain a breakpoint or SNV to be duplicated only if its corresponding genome segment is duplicated. We define another auxiliary variable matrix $D \in \{0,1\}^{l+g}$, where $D_b = 1$ if the breakpoint of SNV b belong to the first allele and 0 otherwise. We also define another auxiliary variable $\Gamma \in \mathbb{Z}_{\geq 0}^{n\times(l+g)\times 2}$, where $\gamma_{i,b,0}$ denotes the segmental copy number of the first allele at the position of the breakpoint or SNV b at node i and $\gamma_{i,b,1}$ is that of the second allele. We impose constraint to ensure that for any existing breakpoint or SNV in the clone, the breakpoint copy number change should be smaller than or equal to the corresponding copy number change at the breakpoint's or SNV's position, except for the newly introduced SNV/SV mutations in the branch. To ensure this, we have the following constraints:

$$\gamma_{j,b,0} - \gamma_{i,b,0} \geq C_{j,b} - C_{i,b} - (2 - E_{i,j} - D_b + W_{i,j,b})(2c_{\max} + 1) \tag{S16}$$

$$\gamma_{j,b,0} - \gamma_{i,b,0} \leq C_{j,b} - C_{i,b} + (2 - E_{i,j} - D_b + W_{i,j,b})(2c_{\max} + 2) \tag{S17}$$

$$\gamma_{j,b,1} - \gamma_{i,b,1} \geq C_{j,b} - C_{i,b} - (1 - E_{i,j} + D_b + W_{i,j,b})(2c_{\max} + 1) \tag{S18}$$

$$\gamma_{j,b,1} - \gamma_{i,b,1} \leq C_{j,b} - C_{i,b} + (1 - E_{i,j} + D_b + W_{i,j,b})(2c_{\max} + 2) \tag{S19}$$

Structural variant and segmental consistency

As described in the main text, we impose that a breakpoint copy number should not be larger than the corresponding segmental copy number. Equation 21 represents the constraints for this. The ratio of the mixed copy numbers of a breakpoint/SNV b and of a segment i is calculated as Equation 22 with the variable $\pi_{i,b}$. To maintain the structural variants and segmental consistency, we set a regularization term to minimize the absolute difference of the estimated and data ratio terms as below:

$$\left| \pi_{i,b} - \frac{\sum_{j=1}^{n} U_{i,j} C_{j,b}}{\sum_{j=1}^{n} U_{i,j}(\gamma_{j,b,0} + \gamma_{j,b,1})} \right| \tag{S20}$$

To represent this with linear constraints, we introduce another auxiliary variable $\delta_{i,b}$, where $i \in \{1, \cdots, n\}$ and $j \in 1, \cdots, l$, and impose the following constraints.

$$\delta_{i,b} \geq \pi_{i,b} \sum_{j=1}^{n} U_{i,j}(\gamma_{j,b,0} + \gamma_{j,b,1}) - \sum_{j=1}^{n} U_{i,j} C_{j,b} \tag{S21}$$

$$\delta_{i,b} \leq -\pi_{i,b} \sum_{j=1}^{n} U_{i,j}(\gamma_{j,b,0} + \gamma_{j,b,1}) + \sum_{j=1}^{n} U_{i,j} C_{j,b} \tag{S22}$$

Using these, we define the last regularization term S, of our objective function, which represents the cost of disagreement between the segmental copy numbers and the copy numbers at the SNV/SV positions.

$$S = \sum_{i=1}^{m}\sum_{b=1}^{l+g} \delta_{i,b} \tag{S23}$$

Inferring Missing Ancestral Clones

We impose constraints that allow TUSV-INT to estimate ancestral clones for which neither DNA nor RNA data are available. This functionality can be used with the `-anc` flag in our framework. We introduce a new binary variable Anc[j] for each clone j, indicating whether clone j is absent from the bulk samples. Next, we impose Eqn. S25 on the clonal frequency matrix U to prevent the model from assigning nonzero frequencies to clones that are not observed in the bulk data.

$$\text{Anc}[j] = \begin{cases} 1 & \text{if clone } j \text{ is absent from the bulk samples} \\ 0 & \text{otherwise} \end{cases} \tag{S24}$$

$$U[i,j] \leq 1 - \text{Anc}[j] \quad \forall i,j \in \{1, \cdots, n\} \tag{S25}$$

To encourage each DNA clone to be assigned to its most closely associated RNA clone, we remove the constraint in Equation 5.

Mapping unsampled SNVs and SVs to the tree

To improve scalability, TUSV-INT first subsamples SNVs and SVs before solving the ILP. Once the reduced ILP has been solved to optimality, we then reassign all of the skipped variants back to the inferred clones using Algorithm S1. It assigns each unsampled SNV or SV to a node in the phylogeny based on the allele-specific copy number of the corresponding genomic segment and observed bulk clonal mixture data. For each variant, we evaluate all non-root nodes and compute two possible clonal frequency estimates depending on i) whether the variant occurred before or ii) after a CNA event. If the variant occurs before the CNA, its copy number is equal to that of the segment in which it lies ($F^{\text{un}}_{i,j,1}$ in Algorithm S1). Conversely, if the variant occurs after the CNA, its copy number is assumed to be one ($F^{\text{un}}_{i,j,2}$ in Algorithm S1). In both cases, the estimated clonal frequency is compared to the observed bulk frequency, and the variant is assigned to the node and scenario that minimize the absolute difference.

Algorithm S1 Map Unsampled SNVs and SVs to Nodes

Input:

E – Adjacency matrix
$A \in \mathbb{R}^{n \times n}$ – Ancestry matrix
$C \in \mathbb{R}^{n \times (l+g+2r)}$ – Copy-number profiles
$U \in \mathbb{R}^{m \times n}$ – Clonal mixture fraction matrix
$F \in \mathbb{R}^{m \times (l+g)^{\text{un}}}$ – Bulk clonal mixture matrix
$Q \in \{0,1\}^{(l+g)^{\text{un}} \times r}$ – SNV/SV to segment mapping

Output:

$W^{\text{un}} \in \{0,1\}^{n \times (l+g)^{\text{un}}}$ – Node assignment matrix

function SNV_SV_ASSIGN(E, A, C, U, F, Q)
  $C1^{\text{un}} \leftarrow C_{:,\, l+g:l+g+r} \cdot Q^\top$
  $C2^{\text{un}} \leftarrow C_{:,\, l+g+r:l+g+2r} \cdot Q^\top$
  $C1^{\text{parent}} \leftarrow E^\top \cdot C1^{\text{un}}$
  $C2^{\text{parent}} \leftarrow E^\top \cdot C2^{\text{un}}$
  ▷ (For the major allele)
  for $j = 1$ to $(l+g)^{\text{un}}$ **do**
    for $i = 1$ to n **do**
      if $C1^{\text{un}}_{i,j} = 1$ **or** $C1^{\text{un}}_{i,j} - C1^{\text{parent}}_{i,j} > 1$ **then**
        $F^{\text{un}}_{i,j,1} \leftarrow U^\top_{:,i} \cdot C1^{\text{un}}_{:,j} + (UA)_{i,:} \cdot C1^{\text{un}}_{:,j}$
      else
        $F^{\text{un}}_{i,j,1} \leftarrow \infty$
      end if
      if $C1^{\text{un}}_{i,j} > 1$ **then**
        $F^{\text{un}}_{i,j,2} \leftarrow U^\top_{:,i} \cdot \mathbf{1} + (UA)_{i,:} \cdot \left(\frac{C1^{\text{un}}_{:,j}}{C1^{\text{un}}_{i,j}} \right)$
      else
        $F^{\text{un}}_{i,j,2} \leftarrow \infty$
      end if
    end for
    $(i^{\text{opt}}_j, d^{\text{opt}}_j) \leftarrow \arg\min_{i \in \{1,\ldots,n\}} \min \left\{ F^{\text{un}}_{i,j,1}, F^{\text{un}}_{i,j,2} \right\}$
  end for
  ▷ (Repeat the same for the minor allele)
  $W^{\text{un}} \leftarrow \mathbf{0}^{n \times (l+g)^{\text{un}}}$
  for $j = 1$ to $(l+g)^{\text{un}}$ **do**
    $W^{\text{un}}[i^{\text{opt}}_j, j] \leftarrow 1$
  end for
  return W^{un}
end function

Deriving bulk mixture matrix F from real bulk DNA-seq data

We construct the F matrix from real data as follows:

- **CNAs:** We use CNVkit (Talevich et al., 2016) to obtain copy number segments, which correspond to columns

$$j \in \{\, l+g+1, \ldots, l+g+2r \,\}$$

of the F matrix. For each segment in the CNVkit output, we take the total copy number (`cn`) and multiply it by the B-allele frequency (`baf`) to compute the allele–specific copy numbers.

- **SNVs:** We call SNVs using Strelka2 (Kim et al., 2018). Each SNV occupies columns

$$j \in \{\, l+1, \ldots, l+g \,\}$$

of F. We compute the B-allele frequency as the ratio of minor-allele depth (AD) to total depth (TD), then multiply this fraction by the segment's total copy number (from CNVkit) to obtain the mixture copy number for the SNV position.
- **SVs:** Structural variant breakpoints are handled similarly as the SNVs. However, our real-data experiment did not include SVs for the tree inference.

Generating simulated data

We used synthetic data for validation purposes to create test cases of known ground truth. Each simulation represented subclones from a single patient with two samples, using genomic segments from chromosomes 1 and 2. We began by generating a binary tree with n clones with a random topology, starting from a normal diploid cell (the root) with no SNVs or SVs. At each non-root clone, we introduced somatic alterations: SNVs were generated at uniformly random positions, and SVs were generated with lengths sampled from a Poisson distribution with a mean of 5,745,000 base pairs, reflecting the average SV length observed in the TCGA-BRCA cohort (Eaton et al., 2018). Allele-specific CNAs were simulated using a relative probability distribution of 2:1:2:1 for amplifications, inversions, deletions, and translocations, respectively. CNAs were modeled as being associated with SVs when the affected genomic regions were duplicated or deleted. We then simulate clonal frequencies for each mixed tumor sample of the same patient uniformly at random, where the frequencies across clones sum to 1. Finally, for each variant j in each sample m, we compute the mixture fractional copy number (i.e., the F matrix) by multiplying the clonal copy numbers with their corresponding clonal frequencies in sample m. To model sequencing errors, we generate segment-specific read counts using a Poisson distribution with a mean depth of 50. These read counts are used as the trial numbers for a binomial density that generates variant-associated read counts based on variant allele frequencies (VAFs). For each variant, we compute theoretical bulk DNA-seq copy numbers as a weighted sum across subclones based on their respective clonal frequencies. Using these copy numbers, we calculate variant allele frequencies (VAFs) for SNVs and breakpoints, as well as B-allele frequencies (BAFs) for CNAs. For transcriptomic simulations, we generate transcript counts using a Poisson density with a mean of 50 for at least 3 and at most 6 genes per genomic segment from the bulk DNA sequences for each clone. These counts are averaged and normalized for allele-specific single-cell RNA sequencing segment-wise CNA profiles.

Supplementary Results

Validation on the correct inference of the missing ancestral clones

To validate our method for handling missing ancestral clones, we evaluated both TUSV-INT and TUSV-ext on trees with $n \in \{3, 5, 7, 9\}$ clones, where only the leaf clones were present in the bulk DNA samples (for both methods) and in the RNA-seq data (for TUSV-INT). This ensures the methods' ability to predict completely unseen internal clones.

In the first set of results (Figure S3(a)), we compare the root mean squared error (RMSE) between the estimated and ground-truth clonal frequency matrices U for both methods (Figure (a)). The next three panels (Figures S3(b), (c), and (d)) show the RMSE of the predicted variant profiles F, as follows: all variants, SNVs only, and SVs only. These results show that TUSV-INT and TUSV-ext achieve comparable performance in reconstructing ancestral clones that are absent from bulk DNA and scRNA samples.

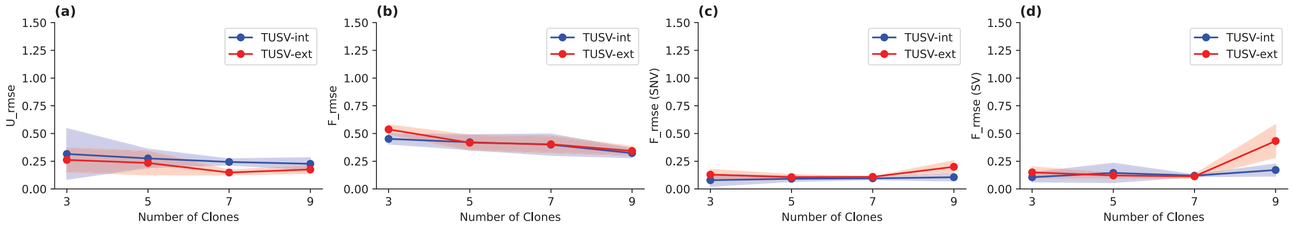

Fig. S3. Results on simulation with missing ancestral clones. (a) shows the root mean squared error of true vs estimated clonal mixture matrix, U. (b) - (d) shows the root mean squared error of true vs estimated bulk mixture copy number matrix, F of TUSV-INT (blue) and TUSV-ext (red). We compared all the variants, only SNVs and only SVs in (b), (c) and (d) respectively.

In the subsequent figure S4, we also compare the estimated copy number matrices C from TUSV-INT and TUSV-ext, with the corresponding true copy number matrices. Here, both methods exhibit higher RMSEs. We hypothesize that this discrepancy arises from the absence of ancestral clones, which may lead to permutations or flips in the ordering of clones (i.e., row assignments in C) when aligning the predicted matrices to the ground truth. This issue is less visible in the F matrix, since it is computed as a matrix product $F = UC$, which is invariant to such child-flipping errors in the clone tree.

Runtime analysis

We ran TUSV-INT on simulated datasets with varying numbers of clones, $n \in \{3, 5, 7, 9\}$, using 10 simulation instances per setting. Each instance was run with 3 iterations, 3 random restarts, and a maximum of 5000 seconds per ILP iteration. The latter restriction is a practical

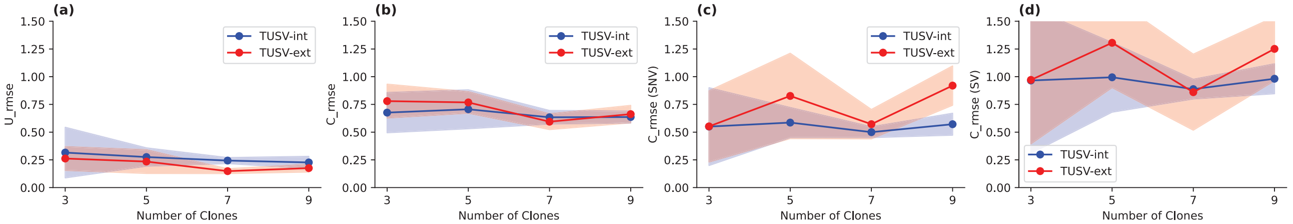

Fig. S4. Results on simulation with missing ancestral clones. (a) shows the root mean squared error of true vs estimated clonal mixture matrix, U. (b) - (d) shows the root mean squared error of true vs estimated variant copy number matrix, C, of TUSV-INT (blue) and TUSV-ext (red). We compared all the variants, only SNVs and only SVs in (b), (c) and (d) respectively.

tradeoff to make the method usable for even difficult instances, capping runtime but at the cost of likely suboptimal solutions when runtime would be excessive. However, it does complicate analysis of runtime. Each simulation included an upper bound of 80 SVs, 40 SNVs, and all genomic segments for CNAs. The total expected runtime per instance was approximately 45,000 seconds, excluding preprocessing time. As shown in Figure S5, TUSV-INT converged more quickly for datasets with fewer clones, often terminating the ILP iterations well before the 5000 second time limit. By contrast, for datasets with higher clone counts, the algorithm utilized the full 5000 seconds allowed per iteration.

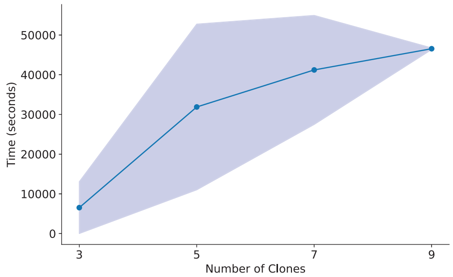

Fig. S5. Runtime per ILP iteration for TUSV-INT on simulations with $n \in \{3, 5, 7, 9\}$ clones with 10 simulation instances, 3, random restarts, 3 iterations, and 5000s time limit. Instances with 3 and 5 clones converge well before the 5000s cutoff, whereas those with 7 and 9 clones uses the full 5000s per iteration.

Convergence analysis

To evaluate convergence across problem sizes, we recorded Gurobi's integrality gap at each branch-and-bound iteration, defined for a minimization problem as:

$$\text{Gap} = \frac{S_{\text{UB}} - S_{\text{LB}}}{S_{\text{LB}}} * 100\%,$$

where S_{UB} is the objective of the best integer solution found so far, and S_{LB} is the best bound produced by the LP relaxations. We ran each simulation for DNA clone counts $n \in \{3, 5, 7, 9\}$ with 3 iterations, 3 random restarts, and 5000 second per ILP iteration. In Figure S6, we plot the minimum integrality gap achieved over all iterations versus the number of clones. A decreasing gap indicates that the solver is closing the distance between the LP relaxation and the incumbent integer solution (where, a 0% gap means Gurobi has found the optimal solution), whereas higher gap means the solution is still far from the optimal one. For 3 and 5 clones, Gurobi drives the gap to near zero well before the time limit; for 7 and 9 clones, it uses the 5000 second time limit and terminates with nonzero gaps, showing that larger instances would require more computational time to converge to explore the larger solution space.

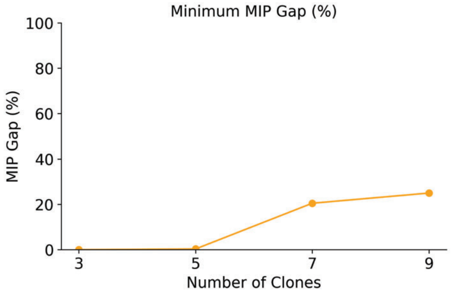

Fig. S6. The minimum MIP gap for the MIP formulation solved by Gurobi vs. the number of clones, $n \in \{3, 5, 7, 9\}$, with 5000s per-iteration time limit. Instances with 3–5 clones reach 0 % MIP gap well before the 5000s limit, while those with 7–9 clones terminate with higher MIP gaps.
